# Similar promotion of Aβ_1-42 _fibrillogenesis by native apolipoprotein E ε3 and ε4 isoforms

**DOI:** 10.1186/1742-2094-1-15

**Published:** 2004-08-16

**Authors:** David Sweeney, Ralph Martins, Harry LeVine, Jonathan D Smith, Sam Gandy

**Affiliations:** 1 Cornell University Medical College, 1300 York Ave, Room A569, New York, NY 10021 USA; 2 Sir James McCusker Alzheimer's Unit, The University of Western Australia, Perth WA, Australia 6009; 3 Sanders-Brown Institute on Aging, University of Kentucky, Lexington, KY 40508 USA; 4 Cleveland Clinic, Cleveland, OH 44195 USA; 5 Farber Institute for Neurosciences Thomas Jefferson University 900 Walnut Street, JHN 467 Philadelphia, PA 19107-5587 USA

## Abstract

The apolipoprotein E ε4 allele contributes to the genetic susceptibility underlying a large proportion (~40–60%) of typical, sporadic Alzheimer disease. Apolipoprotein E deficient mice made transgenic for human apolipoprotein E ε4 accumulate excess cerebral amyloid when compared to similarly prepared mice expressing human apolipoprotein E ε3. Therefore, it is important to search for relevant interactions(s) between apolipoprotein E ε4 and Aβ in order to clarify the biological role for apolipoprotein E ε4 in Alzheimer disease. Using a thioflavine T (ThT)-based assay, we have investigated the effects of native human apolipoprotein E isoforms on the kinetics of Aβ fibrillogenesis. No obvious profibrillogenic activity was detected in Aβ_1-40_-based assays of any native apolipoprotein E isoform. However, when ThT assays were repeated using Aβ_1-42_, modest, but statistically significant, profibrillogenic activity was detected in both apolipoprotein E ε3- and apolipoprotein E ε4-containing media and was similar in magnitude for the two isoforms. These data demonstrate that native apolipoprotein E possesses "pathological chaperone"-type activity for Aβ: in other words, the data indicate that a chaperone-like misfolding reaction can occur between native apolipoprotein E and Aβ. However, the equipotent activities of the apolipoprotein E ε3 and ε4 isoforms suggests the possibility that either extended co-incubation of apolipoprotein E and Aβ, or, perhaps, the inclusion in the reaction of other fibrillogenesis-modulation co-factors (such as metal ions, or inflammatory mediators such as reactive oxygen species, α_2_-macroglobulin, apolipoprotein J, etc.) may be required for modeling *in vitro *the apolipoprotein E-isoform-specific-regulation of extracellular Aβ accumulation that occurs *in vivo*. Alternatively, other events, such as differential apolipoprotein E-isoform-mediated clearance of Aβ or of apolipoprotein E/Aβ complexes may underlie apolipoprotein E-isoform-dependent Aβ accumulation.

## Background

Genetic-neuropathological correlation indicates that the apolipoprotein E type ε4 isoform specifies increased cerebral [1,2] and cerebrovascular [3] accumulation of amyloid β-protein (Aβ). In addition, the apolipoprotein E ε2 isoform can apparently prevent the expression of clinical Alzheimer-type dementia which is otherwise typically associated with amyloidogenic mutations in the amyloid-β protein precursor [4]. Since the apolipoprotein E ε4 allele contributes to the genetic susceptibility underlying a large proportion (~40–60%) of typical, sporadic Alzheimer disease, it is important to search for relevant interactions(s) between apolipoprotein E ε4 and Aβ in order to clarify the biological role for apolipoprotein E ε4 in Alzheimer disease. Currently proposed mechanisms include differential activities of apolipoprotein E isoforms in modulating Aβ fibrillogenesis [5-7] and/or Aβ clearance [8,9]. Many studies of apolipoprotein E modulation of Aβ fibrillogenesis have utilized denatured apolipoprotein E, purified from the serum of human apolipoprotein E homozgotes following extraction in organic solvents [10]. While providing a convenient source of pure apolipoprotein E protein, this preparation does not represent native apolipoprotein E as it exists *in vivo*. Using a thioflavine T (ThT)-based assay [11] we have investigated the effects of native human apolipoprotein E isoforms on the kinetics of Aβ fibrillogenesis.

## Methods

Synthetic Aβ_1-40 _or Aβ_1-42 _(Keck Foundation Protein Facility, Yale University, New Haven CT) was freshly prepared for each assay at a concentration of 16 mg/ml in distilled, deionized water and diluted with phosphate-buffered saline (PBS) to generate a 5 mg/ml working solution. The "aggregation step" consisted of a reaction mixture containing 8 μl Aβ peptide (1 mg/ml final conc) and 32 μl of either apolipoprotein E ε3- or ε4-containing conditioned medium or control conditioned medium from SV40 empty vector-transfected cells.

For the investigation of native apolipoprotein E preparations, apolipoprotein E isoforms were generated in the conditioned medium of stably-transfected SV40-apolipoprotein E ε3-, or SV40-apolipoprotein E ε4-, expressing Chinese hamster ovary (CHO) cells (CHO cells lack detectable endogenous apolipoprotein E; data not shown). All conditioned media were prepared using Dulbecco's minimal essential medium supplemented with 0.2% (wt/vol) bovine serum albumin only (no fetal bovine serum). Apolipoprotein E isoform levels were determined by quantitative immunoblotting of conditioned medium and apolipoprotein E-containing serum standards, the latter having been kindly provided by Dr. Petar Alaupovic of the Oklahoma Medical Research Foundation (Oklahoma City OK). Conditioned medium apolipoprotein E concentrations were then standardized using control medium conditioned by SV40 empty vector-transfected cells as diluent, yielding a final concentration of apolipoprotein E of 14 μg/ml, within the range of that reported in human cerebrospinal fluid. Since the final concentration of Aβ peptide was 1 mg/ml, the Aβ/apolipoprotein E stoichiometry (molar ratio) was ~500:1, suggesting models for the Aβ/apolipoprotein E interaction based either on a "catalytic" "pathological chaperoning" model of apolipoprotein E action on Aβ, or with a "seeding" model of Aβ folding. Detailed biochemical characterization of this native apolipoprotein E preparation has been reported [9].

The "aggregation step" fibrillogenesis reaction [11] was incubated at 37°C until the time of the ThT fluorescence measurement, which was performed from 1 to 7 days later. For the "measurement step" [11], 960 μ1 of 10 μM ThT (Nakarai Chemicals, Kyoto, Japan) in 50 mM phosphate buffer (pH 6.0) was added to the "aggregation step" reaction mixture. Within 30 minutes after addition of ThT, fluorescence was measured with a Millipore Cytofluor (Bedford MA) in each of five successive 200 μl aliquots of the reaction mixture, using an excitation filter of 450 nm and an emission filter of 482 nm, and a temperature of 25°C.

In order to standardize the ThT assay in our Laboratory, we performed studies of Aβ fibrillogenesis following 1–7 day incubations of Aβ_1-40_, either in physiological phosphate buffer alone or in the presence of metal ions (Zn^2+^, Fe^2+^, or A1^3+^; 1 mM final conc [12]. ThT fluorescence and ultrastructural features were measured daily (not shown). Profibrillogenic activities of the metal ions tested were in agreement with a published report [12] (e.g., Al^3+^stimulated ThT fluorescence of Aβ_1-40 _by 3.6 ± 1.1- to 5.7 ± 1.4-fold; p < 0.01), indicating that our Aβ preparations were capable of metal ion-induced fibrillogenesis. Metals were not present during assessment of profibrillogenic effects of apolipoprotein E isoforms.

## Results and discussion

No obvious profibrillogenic activity was detected in Aβ_1-40_-based assays of any native apolipoprotein E isoform (Table 1). However, when ThT assays were repeated using Aβ_1-42_, modest, but statistically significant, profibrillogenic activity was detected in both apolipoprotein E ε3- and apolipoprotein E ε4-containing media and was similar in magnitude for the two isoforms (Table 1). The observation of a profibrillogenic effect of apolipoprotein E specifically for Aβ_1-42 _has been noted [5] and is of particular interest in light of biophysical and molecular neuropathological evidence suggesting that "long" Aβ peptides ending at positions N-42 or N-43 are apparently crucial for the initiation ("seeding") of Aβ deposition [13].

**Table 1:** Effects of native apolipoprotein E isoforms on fibrillogenesis of Aβ_1-40 _and Aβ_1-42_. Fold-effects represent means ± SEM of the quotients of ThT fluorescence values obtained for each Aβ peptide in the presence of apolipoprotein E-isoform-containing conditioned medium divided by ThT values obtained in the presence of conditioned medium lacking apolipoprotein E, derived from empty vector-transfected cells (n = 5–6).

Aβ_1-40_			
1 day co-incubation	CHO apolipoprotein E ε3	1.0 ± 0.l-fold	N.S.
	CHO apolipoprotein E ε4	1.0 ± 0.l-fold	N.S.
7 day co-incubation	CHO apolipoprotein E ε3	1.0 ± 0.l-fold	N.S.
	CHO apolipoprotein E ε4	1.2 ± 0.l-fold	N.S.
Aβ_1-42_			
4 day co-incubation	CHO apolipoprotein E ε3	1.7 ± 0.27-fold	p < 0.01
	CHO apolipoprotein E ε4	1.6 ± 0.18-fold	p < 0.005
7 day co-incubation	CHO apolipoprotein E ε3	1.7 ± 0.22-fold	p < 0.005
	CHO apolipoprotein E ε4	1.8 ± 0.19-fold	p < 0.0005

These data demonstrate that native apolipoprotein E possesses "pathological chaperone"-type activity for Aβ: in other words, the data indicate that a chaperone-like misfolding reaction can occur between native apolipoprotein E and Aβ, at least at the concentrations and proportions evaluated herein. However, the equipotent activities of the apolipoprotein E ε3 and ε4 isoforms suggests the possibility that either extended co-incubation of apolipoprotein E and Aβ, or, perhaps, the inclusion in the reaction of other fibrillogenesis-modulation co-factors (such as metal ions, or inflammatory mediators such as reactive oxygen species, α_1_-antichymotrypsin, heparin sulfate-protcoglycan, non-Aβ component, apolipoprotein J, complement, etc.) may be required for modeling *in vitro *the apolipoprotein E-isoform-specific-regulation of extracellular Aβ accumulation that occurs *in vivo*.

Alternatively, other events, such as differential apolipoprotein E-isoform-mediated clearance of Aβ or of apolipoprotein E/Aβ complexes [8,9,14] may contribute to apolipoprotein E-isoform-dependent Aβ accumulation. Differential anti-inflammatory activity might also play a role. Further investigation will be required in order to elucidate the precise mechanism(s) which specify how apolipoprotein E ε4 promotes Aβ accumulation in human brain and cerebral vessels *in vivo*.

## List of abbreviations

ThT, thioflavine T; Aβ, amyloid-β peptide; PBS, phosphate-buffered saline; CHO, Chinese hamster ovary cells.

## Competing interests

None declared.

## Authors' contributions

DS performed all assays, including the ThT assay, which was originated by HL. HL also oversaw the transfer of the assay from his lab to ours. RM prepared standard conditioned media from transfected cells provided by JDS. SG oversaw the project, supported the project as noted below, and wrote the manuscript.
